# Plk2-mediated phosphorylation and translocalization of Nrf2 activates anti-inflammation through p53/Plk2/p21^cip1^ signaling in acute kidney injury

**DOI:** 10.1007/s10565-022-09741-1

**Published:** 2022-07-16

**Authors:** Da-Eun Kim, Hye Eun Byeon, Dae-Hoon Kim, Sang Geon Kim, Hyungshin Yim

**Affiliations:** 1 Department of Pharmacy, College of Pharmacy, Institute of Pharmaceutical Science and Technology, Hanyang University, Ansan, 15588 Gyeonggi-do Korea; 2 College of Pharmacy and Integrated Research Institute for Drug Development, Dongguk University-Seoul, Goyang-si, 10326 Gyeonggi-Do Korea; 3 College of Pharmacy, Seoul National University, Gwanakro 599, Seoul, 08826 Korea

**Keywords:** Plk2, Nrf2, p53, Anti-inflammation, Acute kidney injury

## Abstract

The Plk2 is a cellular stress-responsive factor that is induced in response to oxidative stress. However, the roles of Plk2 in acute kidney injury (AKI) have not been clarified. We previously found that Plk2 is an interacting factor of Nrf2 in response to cellular stress, since Plk2 is upregulated in the Nrf2-dependent network. Here, we show that the levels of p53, Plk2, p21^cip1^, and chromatin-bound Nrf2 were all upregulated in kidney tissues of mice or NRK52E cells treated with either cisplatin or methotrexate. Upregulation of Plk2 by p53 led to an increase of Nrf2 in both soluble and chromatin fractions in cisplatin-treated NRK52E cells. Consistently, depletion of Plk2 suppressed the levels of Nrf2. Of note, Plk2 directly phosphorylated Nrf2 at Ser40, which facilitated its interaction with p21^cip1^ and translocation into the nuclei for the activation of anti-oxidative and anti-inflammatory factors in response to AKI. Together, these findings suggest that Plk2 may serve as an anti-oxidative and anti-inflammatory regulator through the phosphorylation and activation of Nrf2 to protect kidney cells from kidney toxicants and that Plk2 and Nrf2 therefore work cooperatively for the protection and survival of kidney cells from harmful stresses.

**Supplementary Information:**

The online version contains supplementary material available at 10.1007/s10565-022-09741-1.

## Introduction

Acute kidney injury (AKI), which is characterized by a rapid decrease in renal function together with the accumulation of toxic metabolic products such as oxidative byproducts, is one of the major adverse effects of several chemotherapeutic anticancer agents (Lameire 2014; Salahudeen et al. 2013). The incidence of AKI in patients with cancer is at least three-fold higher than that of patients with other diseases (Lameire 2014; Salahudeen et al. 2013). The development of AKI in cancer patients in response to chemotherapeutics has limited the effectiveness and selection of medication treatment. Moreover, the presence of AKI negatively affects the survival of patients by increasing the risk of death (Christiansen et al. 2011). Because of the high frequency of enhanced oxidative stress in AKI, a better understanding of the pathogenesis of AKI triggered by anticancer agents and the identification of mediators that suppress AKI development are crucial to prevent AKI and improve survival in cancer patients. It would also facilitate the development of more effective anticancer agents with enhanced clinical outcomes.

Polo-like kinase 2 (Plk2), a member of the polo-like kinase family containing polo-box domains and a kinase domain (Kim et al. 2015; Lowery et al. 2005), was originally identified as an immediate-early response gene and may play a role in the protection and survival of the cell from cellular stress and DNA damage (Ma et al. 2003b). A previous study showed that Plk2-/- mice are viable although growth was delayed, and Plk2-/- cells showed delayed entry into S phase (Ma et al. 2003a), being consistent with the observation that Plk2 is essential for centriole duplication in S phase (Cizmecioglu et al. 2012; Krause and Hoffmann 2010; Warnke et al. 2004). In addition, Plk2 is crucial for cell survival with defective mitochondrial respiration (Matsumoto et al. 2009). Plk2 has also been recognized as a tumor suppressor in several cancers (Burns et al. 2003; Coley et al. 2012; Matthew et al. 2007; Pellegrino et al. 2010; Smith et al. 2006; Zhao et al. 2015). However, in colorectal, bladder, and pancreatic cancer, Plk2 was reported as an oncogenic factor because of its overexpression and tumor-promoting effects (Kothari et al. 2013; Ou et al. 2016; Tan et al. 2010). These results indicate that Plk2 function in carcinogenesis may depend on tissue-specific characteristics. Most studies related to Plk2 have focused on defense mechanisms against oxidative stress (Li et al. 2014). Thus, Plk2 expression is directly upregulated by p53 in paclitaxel-treated cells (Burns et al. 2003), whereas Plk2 depletion increases cell death in aphidicolin-induced S phase arrest (Matthew et al. 2007). We recently found that Plk2 was highly upregulated in response to weak damage induced by doxorubicin in hepatocellular carcinoma (Joo et al., 2019). Based on these previous studies, we hypothesized that Plk2 may play a pivotal role in the defense from oxidative stress and DNA damage in AKI.

Nuclear factor erythroid 2–related factor 2 (Nrf2) is one of the most critical protective factors against cellular oxidative stress. Nrf2 regulates the transcription of a series of genes involved in antioxidant defense such as genes encoding superoxide dismutase, heme oxygenase-1, and catalase through its binding to the antioxidant response elements (AREs) in the promoter regions of target genes (Kobayashi and Yamamoto 2006; Ma 2013). Nrf2 is maintained at low levels by the interaction with Keap1, an adaptor protein of the cullin 3–ubiquitin E3 ligase complex, which accelerates its proteasomal degradation through ubiquitination (Itoh et al., 2003; Kobayashi et al. 2009; McMahon et al. 2003). In addition to Keap1, several other proteins have been shown to regulate Nrf2 including the p53 target p21^cip1^ and autophagy substrate p62 (Chen et al. 2009; Komatsu et al. 2010). Nrf2 is critical for preventing renal toxicity induced by cisplatin through transcriptional activation of microRNA-125b (Joo et al. 2013). Recently, we found that an Nrf2-activating lncRNA functions as a cell fate regulator by p53-dependent Nrf2 activation and that Plk2 forms a complex with Nrf2 under survival conditions from oxidative stress in hepatocellular cancer (Joo et al. 2019). Nonetheless, whether and how Plk2 and Nrf2 protect kidney cells from AKI remains unclear.

Here, we report that in response to AKI in the kidney, the activation of Atm and p53 induces Plk2 and p21^cip1^, which then activates Nrf2 through phosphorylation and recruitment of Nrf2 into the nuclei for the expression of anti-inflammatory factors as well as anti-oxidative regulators.

## Materials and methods

### Materials

Human embryonic kidney HEK293 cells and rat kidney proximal tubular epithelial NRK52E cells were purchased from ATCC (Manassas, VA, USA). Dulbecco’s modified Eagle’s medium (DMEM), fetal bovine serum (FBS), calf serum, streptomycin, and penicillin were purchased from Corning Cellgro (Manassas, VA, USA). Cisplatin was purchased from Millipore (Burlington, MA, USA). All other chemical reagents including methotrexate were purchased from Sigma-Aldrich (St. Louis, MO, USA).

### Cell culture and treatment

NRK52E and HEK293 cells were cultured in DMEM (Corning) supplemented with 10% calf serum and FBS, respectively, with antibiotics. Cells were cultured in a 5% CO_2_ incubator at 37 °C. For the cisplatin or methotrexate treatment, NRK52E cells were seeded at 3x10^4^ cells/ml, and after 24 h, the cells were treated with cisplatin or methotrexate during the indicated periods.

### Animal treatment

Nine-week-old male C57BL/6 mice (Samtako Company, Osan, Korea) were injected with cisplatin (at a single dose of 15 mg/kg body weight), and the cortices of the kidney were prepared for biochemical experiments. Nrf2 knockout (KO) C57BL/6 mice were used with only male mice and at least 6 months. Three days after injected with 15 mg/kg cisplatin, the cortices of kidney tissues in wild type and KO of Nrf2 mice were obtained. Animal experiments for this study were regulated and managed by the guidelines and ethical approvals of the Institutional Animal Care and Use Committee (IACUC) at Seoul National University (Lee et al. 2014).

### Lentivirus-based shRNA preparation

We prepared lentivirus-based shRNA-expressing plasmids targeting rat *Plk2* (gene access no. NM_031821.2) at 232–252 bp (CGCTACTGCCGGGGCAAAGTG) (pLKO-Puro.1-rPlk2) and rat *Tp53* (gene access no. NM_030989.3) at 1013–1033 bp (TGTTCCGAGAGCTGAATGAGG) (pLKO-Puro.1-rTp53) for the loss of function experiments. Lentivirus was generated as described previously (Jang et al. 2021; Yim and Erikson 2009). Cells were infected and selected using puromycin for 3 days.

### Immunoblot analysis

Cell lysates were extracted in lysis buffer [20 mM Tris (pH 7.5), 2 mM MgCl_2_, 1 mM EGTA, 1 mM dithiothreitol (DTT), 0.5% Triton X-100, 50 mM β-glycerophosphate, 25 mM NaF, 1 mM sodium vanadate, 100 mg/ml PMSF, and protease inhibitor cocktail (Roche; Indianapolis, IN, USA)], as described previously (Jang et al. 2021). For the chromatin fraction, the pellets were sonicated in CSK buffer [0.5% Triton X-100, 10 mM PIPES (pH 6.8), 100 mM NaCl, 300 mM sucrose, 3 mM MgCl_2_, 1 mM EGTA, 1 mM DTT, 1 mM phenylmethylsulfonyl fluoride (PMSF), 50 mM NaF, 0.1 mM sodium vanadate, and protease inhibitor cocktail (Roche)], and then, chromatin was incubated with 50U benzonase at 37℃ for 10 min, as described previously (Yim and Erikson 2009). Equal amounts of protein were separated by SDS-PAGE, and immunoblot analysis was performed with the indicated antibodies as follows: Plk2 (Abcam, ab34811), Nrf2 (Santa Cruz Biotechnology, sc-13032), phospho-S40-Nrf2 (Bioss, bs-2013R), p53 (Santa Cruz Biotechnology, sc-393031), phospho-S15-p53 (Santa Cruz Biotechnology, sc11764), Plk1 (Millipore, 05–844), phospho-S1981-Atm (Calbiochem, DR1003), Atm (Santa Cruz Biotechnology, sc-23921), p21^cip1^ (Santa Cruz Biotechnology, sc-6246), Puma (Cell Signaling Technology, 4976), Mdm2 (Santa Cruz Biotechnology, sc-965), IL4 (Santa Cruz Biotechnology, sc-53084), IL10 (Santa Cruz Biotechnology, sc-365858), Histone H1 (Santa Cruz Biotechnology, sc-8030), α-tubulin (Sigma, T6074), Flag (Sigma, F3165), and β-actin (Sigma, A5441). The protein bands were displayed, and the intensity values were determined using an Odyssey infrared imaging system (LI-COR Biosciences; Lincoln, NE, USA).

### Immunoprecipitation assay

Cell lysates were incubated with normal IgG (Santa Cruz Biotechnology, sc-2027), anti-Flag (Sigma, F3165), or anti-Nrf2 (Santa Cruz Biotechnology, sc-13032) antibodies for 18 h at 4°C with end-over-end mixing, followed by incubation with protein A/G agarose (Santa Cruz Biotechnology, sc-2002) for 4 h at 4°C. Samples were centrifuged, supernatants were removed, and immunoprecipitates were washed three times with lysis buffer. Samples were resolved by SDS-PAGE and analyzed by immunoblot.

### Quantitative real-time polymerase chain reaction (qRT-PCR)

Total RNA was extracted by Trizol (Invitrogen Life Technologies, Carlsbad, CA, USA). cDNA was synthesized from 1 μg RNA using a First Strand cDNA Synthesis Kit (Thermo Scientific). qRT-PCR was performed with the cDNA, SYBR Green Master Mix (Bio-Rad; Hercules, CA, USA), and gene-specific primers using a CFX96 Real-Time PCR system (Bio-Rad). The primer sequences are shown in Supplementary Table 1.

### Immunofluorescence

NRK52E cells grown on coverslips were fixed with 4% paraformaldehyde. Methanol was used for the permeabilization. Cells were washed three times with 0.1% Triton X-100 in phosphate-buffered saline (PBS), incubated overnight at 4 °C in PBS containing 0.1% Triton X-100 and 3% bovine serum albumin to block nonspecific interactions, and then incubated with anti-Nrf2 (Santa Cruz Biotechnology, sc-13032), Plk2 (Santa Cruz Biotechnology, sc-374643), p21^cip1^ (Santa Cruz Biotechnology, sc-6246), and p21^cip1^ (MyBioSource, San Diego, CA, USA; MBS440016) antibodies. The cells were washed three times with PBST (0.1% Triton X-100) and then incubated with fluorescein isothiocyanate (FITC)–conjugated anti-rabbit secondary antibodies (Invitrogen), fluorescein isothiocyanate (FITC)–conjugated anti-mouse secondary antibodies (Jackson ImmunoResearch Laboratories; West Grove, PA, USA), Cy3-conjugated anti-rabbit secondary antibodies (Jackson ImmunoResearch Laboratories), or Cy3-conjugated anti-mouse secondary antibodies (Jackson ImmunoResearch Laboratories) and 4′, 6-diamidine-2-phenylindole (DAPI) (Sigma-Aldrich) for staining nuclear DNA. Images of cells were collected and evaluated with a confocal microscope FW3000 (Olympus; Tokyo, Japan).

### Bioinformatics analysis

The transcriptome data were obtained from an online gene set database (GSE48879**)** according to the previous report (Joo et al. 2013). The results from the analysis comparing wild-type or Nrf2 KO mice are described as fold change. For analysis, gene probes with significant fold changes (more than 1.5) were clustered. For developing a significant probe list, gene enrichment and functional annotation analysis using Gene Ontology (http://geneontology.org/) and KEGG (http://kegg.jp) was performed.

### Statistical analysis

Data are shown as the means ± SDs of at least three independent experiments. Statistical significances were analyzed using student’s *t*-test, and statistical significance was set at *p* < 0.05.

## Results

### Cisplatin-induced AKI increases the expressions of Plk2, p53, and Nrf2, which upregulate the levels of nuclear chromatin-bound Nrf2 in mouse kidneys

**Fig. 1:**
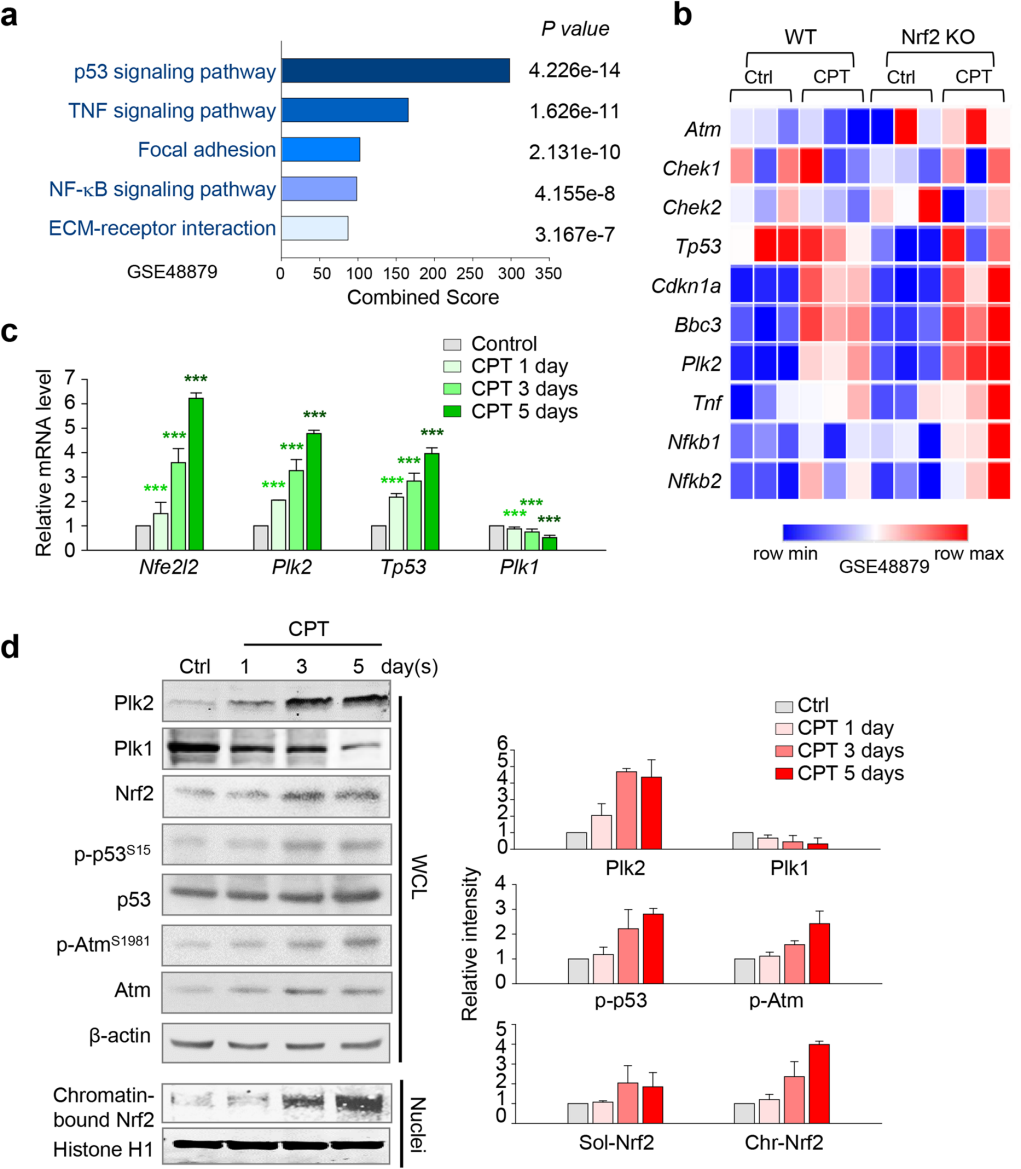
Cisplatin-induced AKI increases the expressions of Plk2, p53, and Nrf2, which upregulate the levels of nuclear chromatin-bound Nrf2 in mouse kidney. **a** The previously published microarray dataset (GSE48879) was analyzed by KEGG pathway analysis. Analysis of transcriptome data for gene probes with fold changes of more than 1.5 (general cutoff point for DEGs) and *p* values are displayed. **b** A heatmap analysis was performed for *Atm*, *Chek1*, *Chek2*, *Tp53*, *Cdkn1a*, *Bbc3*, *Plk2*, *Tnf*, *Nfkb1*, and *Nfkb2* using a published transcriptome dataset (GSE48879) from cisplatin (CPT)–treated mice. **c**–**d** Kidney samples of the mice were taken at 1, 3, and 5 day(s) after a single injection of cisplatin (15 mg/kg, i.p., *n* = 4 or 5 per group). **c** Quantitative RT (qRT)-PCR was performed to determine the mRNA levels of *Plk1*, *Plk2*, *Tp53*, and *Nfe2l2* in the kidney of mice treated with cisplatin for 1, 3, and 5 day(s). The relative expression of each gene was plotted. ***p < 0.001. **d** Immunoblot analysis was performed using the lysates of kidney cortex of the mice injected with cisplatin for 1, 3, and 5 day(s). The expressions of Plk2, Plk1, Nrf2, p-p53 (Ser15), p53, p-Atm (Ser1981), Atm, Histone H1, and β-actin were measured by immunoblottings. Chromatin fractions were isolated from whole-cell lysates, and the expression of Nrf2 protein was analyzed by immunoblot. Histone H1 was used as a loading control for the nuclear fraction

To understand the signaling pathway in cisplatin-induced AKI, KEGG pathway analyses were performed using a previously published microarray dataset (GSE48879), and then, top five pathways were extracted. The p53 signaling pathway was the most relevant pathway in cisplatin-treated Nrf2 knockout (KO) mice (Fig. 1a). The expression levels of DNA damage–related factors such as *Atm*, *Chek1*, *Chek2,* and *Tp53* were observed in the cisplatin-treated mice using the GSE48879 dataset (Fig. 1b). A heatmap showed that DNA damage–sensing kinase *Atm* and its downstream *Chek1* and *Tp53* were upregulated by cisplatin treatment in Nrf2 KO mice compared with control. The levels of *Cdkn1a* and *Bbc3*, transcriptional targets of p53, were also markedly increased in cisplatin-treated mice (Fig. 1b). Moreover, the levels of *Plk2*, a target of p53, were higher in cisplatin-treated mice than those of the control, and this difference was more pronounced in Nrf2 KO mice treated with and without cisplatin (Fig. 1b).

To investigate the regulatory functions of the cellular stress-responsive factor Plk2 and the Nrf2 defense molecule from oxidative stress in AKI, we examined the mRNA levels of *Plk2* and *Nfe2l2* in cisplatin-treated C57BL/6 mice over 5 days (Fig. 1c). Mice were subjected to a single injection of cisplatin (15 mg/kg, i.p.) and sacrificed at indicated days, and kidneys were harvested for analysis. *Plk2* mRNA levels were increased, beginning from day 1 after administration of cisplatin compared with expression in the control. The levels of *Plk2* mRNA were increased approximately five-fold in cisplatin-treated mice at day 5 (Fig. 1c). Similarly, the levels of *Nfe2l2* were approximately six-fold higher at day 5 in cisplatin-treated mice compared with that in the control. However, the levels of Plk1 were markedly reduced in a time-dependent manner after cisplatin treatment (Fig. 1c). Since p53 is known as an upstream factor of Plk2 (Burns et al. 2003), the mRNA levels of p53 were observed; as expected, the mRNA levels of p53 were also upregulated (Fig. 1c). Together, these findings show that the mRNA levels of Plk2, p53, and Nrf2 increased, while Plk1 mRNA was reduced by treatment of cisplatin in mice.

p53 and ATM kinase are major regulators in response to cellular stress and DNA damage (Cha and Yim 2013). Therefore, we examined the protein and phosphorylation levels of Atm and p53 in cisplatin-treated mice (Fig. 1d). The levels of p53 and Atm both were increased in a similar manner as Plk2, in concurrence with the changes of mRNA levels shown in Fig. 1c. Moreover, the phosphorylation of p53 at Ser15 and Atm at Ser1981 increased in a time-dependent manner after the administration of cisplatin. Plk2 increased in a similar manner as the changes in p-p53 and p-Atm, accompanied with an increase of Nrf2 in response to cisplatin-induced damage (Fig. 1d). We further examined levels of Nrf2 in whole-cell lysates and chromatin fractions (Fig. 1d). Chromatin-bound Nrf2 markedly enhanced in a time-dependent manner; at 5 days after cisplatin treatment, it was four-fold higher than that of controls (Fig. 1d). The changes in chromatin-bound Nrf2 levels reflected the total levels of Nrf2 in cisplatin-induced mice (Fig. 1c–d). Thus, cisplatin-induced AKI triggered the upregulation of Plk2, p53, and Nrf2 but not Plk1.

### Cisplatin treatment increases levels of Plk2, p53, and Nrf2 in rat kidney NRK52E cells

**Fig. 2:**
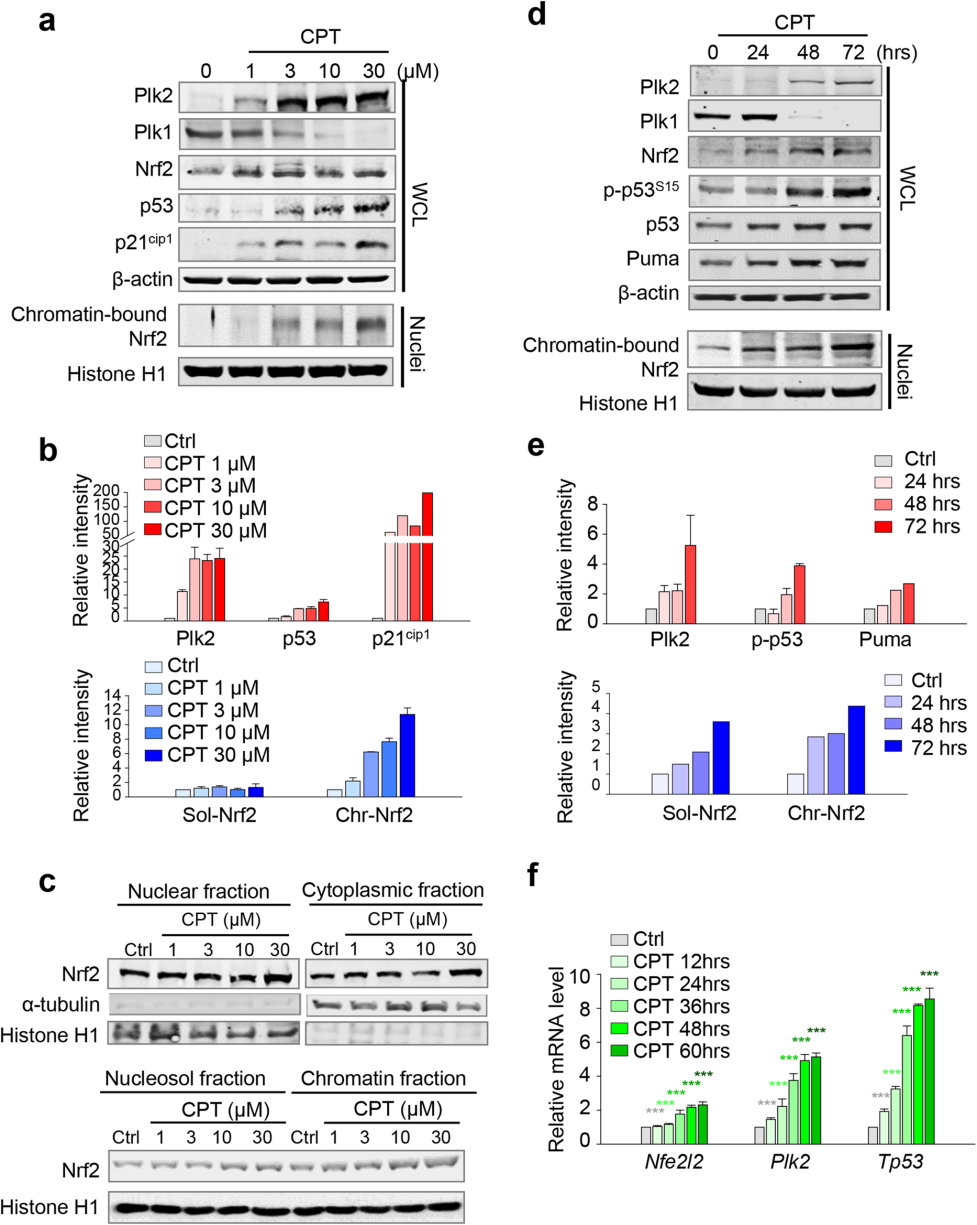
Cisplatin treatment increases levels of Plk2, p53, and Nrf2 in rat kidney NRK52E cells. **a**–**c** Cisplatin (CPT) was treated at various concentrations of NRK52E rat renal proximal tubular cells at the indicated concentrations. **a** Immunoblot was performed with whole lysates using antibodies against Plk1, Plk2, p53, p21^cip1^, Nrf2, Histone H1, and β-actin. For detection of chromatin-bound proteins, Histone H1 was used as a loading control. **b** The relative band intensity values of Plk2, p53, p21^cip1^, soluble Nrf2, and chromatin-bound Nrf2 were measured using LI-COR Odyssey software. **c** A fractionation assay was performed. Nuclear and cytoplasmic fractions were prepared from whole cells; nuclear soluble fraction and chromatin fraction were from the nuclear fraction. Nrf2 protein was analyzed by immunoblot. Histone H1 was used as a loading control of the nuclear fraction. **d**–**e** Cisplatin was treated to NRK52E cells at the indicated times. **d** Immunoblot was performed with whole lysate using antibodies against Plk2, Plk1, p-p53 (S15), p53, Puma, Nrf2, β-actin, and Histone H1. For detection of chromatin-bound proteins, Histone H1 was used as a loading control. **e** The relative band intensity values of Plk2, p-p53, Puma, soluble Nrf2, and chromatin-bound Nrf2 were analyzed using LI-COR Odyssey software. **f** qRT-PCR assays were performed using primers for *Plk2*, *Tp53*, and *Nfe2l2*. ***p < 0.001 compared with control

We next examined the effects of the cisplatin-induced acute injury on the expression of Plk2, p53, and Nrf2 in rat kidney NRK52E cells (Fig. 2). Immunoblot analysis was performed after cisplatin treatment at the indicated concentrations for 48 h in NRK52E cells (Fig. 2a–c) or after 30-μM cisplatin treatment over various times (Fig. 2d–e). The expressions of Plk2, p53, and Nrf2 proteins were all increased together concentration- and time-dependently (Fig. 2a–d). Consistent with the results of AKI of mice, the level of chromatin-bound Nrf2 protein increased in time- and concentration-dependent manners after cisplatin treatment, while the levels of Nrf2 were slightly upregulated (Fig. 2a–e). Additional fractionation assay showed that chromatin-bound Nrf2 was markedly increased compared with the nucleosol and cytoplasmic fractions in a concentration-dependent way in cisplatin-treated NRK52E cells (Fig. 2c). Consistent with the mRNA expression changes shown in Fig. 1c, the mRNA levels of Nrf2, Plk2, and p53 were increased in a time-dependent manner in cisplatin-treated NRK52E cells (Fig. 2f). Together, these findings show that the levels of p53, Plk2, soluble Nrf2, and chromatin-bound Nrf2 were upregulated in a time-dependent or a concentration-dependent manner in response to cisplatin-induced injury of kidney cells.

### Regulation of Plk2 by p53 results in the activation of Nrf2 and its nuclear location in response to cisplatin-induced stress of NRK52E cells

**Fig. 3:**
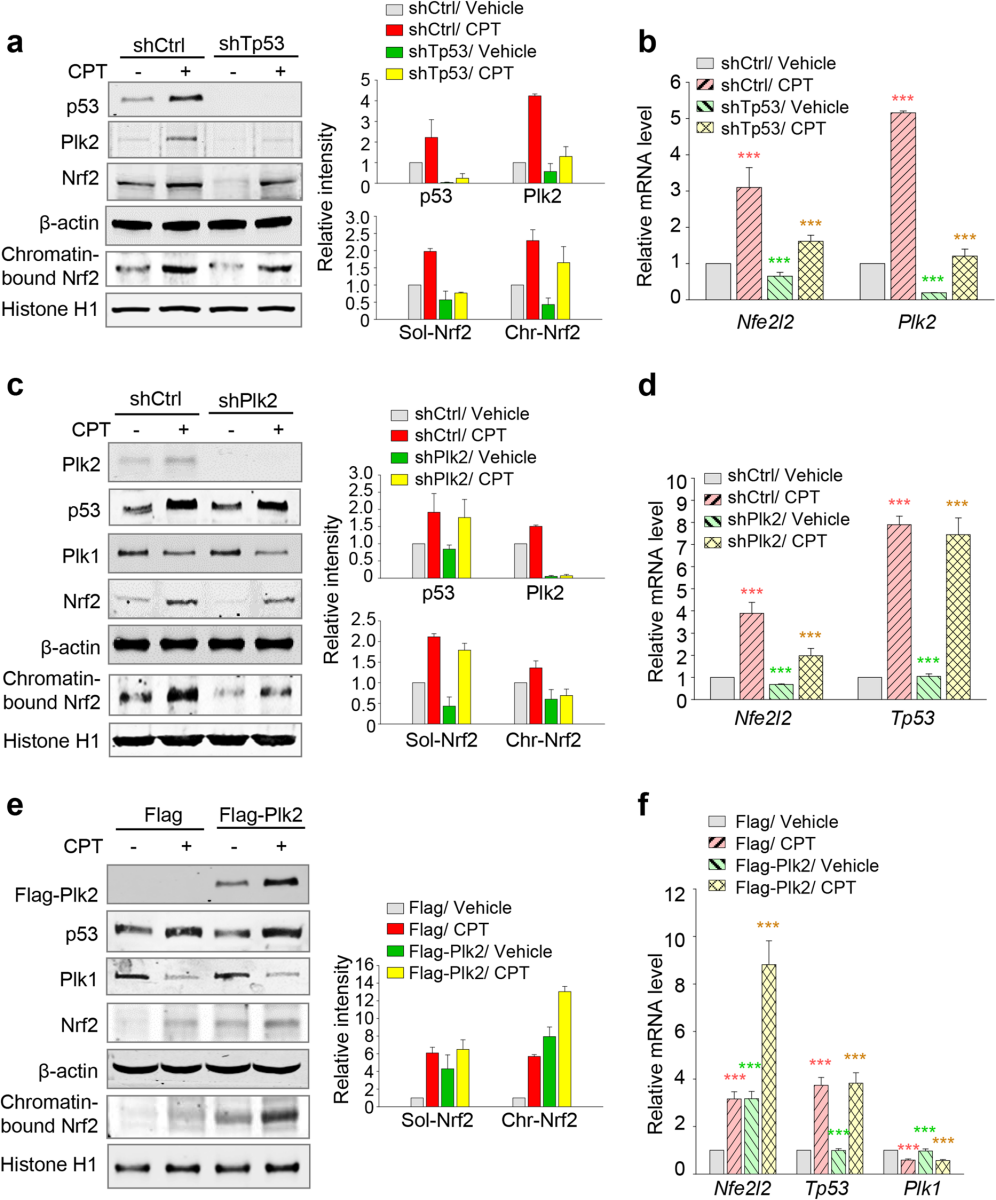
Regulation of Plk2 by p53 results in the activation of Nrf2 in response to cisplatin-treated NRK52E cells. **a**–**b** NRK52E cells were infected with lentiviral shRNA targeting rat *Tp53*. Twenty-four hours after infection, the cells were treated with/without 30 μM cisplatin for 48 h. **a** Immunoblot analysis was performed with antibodies for p53, Plk2, Nrf2, β-actin, and Histone H1. Histone H1 was used as a loading control for the chromatin fraction. The relative band intensity values of p53, Plk2, soluble Nrf2, and chromatin-bound Nrf2 were measured using LI-COR Odyssey software. **b** mRNA was prepared from NRK52E cell lysates for qRT-PCR analysis. The mRNA expression levels of *Plk2* and *Nfe2l2* were determined. ***p < 0.001. **c**–**d** Rat *Plk2* shRNA was introduced in NRK52E cells. Twenty-four hours after infection, the cells were treated with/without 30 μM cisplatin for 48 h. **c** Immunoblot analysis was performed with antibodies for Plk2, p53, Plk1, Nrf2, β-actin, and Histone H1. The relative band intensity values of p53, Plk2, soluble Nrf2, and chromatin-bound Nrf2 were measured. **d** mRNA was prepared from NRK52E cell lysates for qRT-PCR analysis. The mRNA expression levels of *Nfe2l2* and *Tp53* were determined and plotted. ***p < 0.001 compared with vehicle cells treated with control shRNA (shCtrl). **e**–**f** Flag-tagged Plk2 proteins were expressed in NRK52E cells. Twenty-four hours after transfection, 30 μM cisplatin was treated to the cells for 48 h.** e** Immunoblot analysis was performed with antibodies for Flag, p53, Plk1, Nrf2, β-actin, and Histone H1. The relative band intensity values of soluble Nrf2 and chromatin-bound Nrf2 were analyzed using LI-COR Odyssey software. **f** qRT-PCR was performed to evaluate the mRNA levels of rat *Nfe2l2*, *Tp53*, and *Plk1* in NRK52E cells. ***p < 0.001 compared with vehicle cells expressing Flag-mock control

Plk2 is a putative target of p53 in response to cellular stress (Burns et al. 2003). We examined the expressions of Plk2 and Nrf2 in kidney cells depleted for p53 using shRNA targeting rat *Tp53* in the presence or absence of cisplatin (Fig. 3a). Immunoblot analysis showed that the levels of Plk2 were reduced in p53-depleted cells compared with those of control cells (Fig. 3a). In addition, soluble and chromatin-bound Nrf2 levels were lowered by knockdown of p53 compared with those of control cells in cisplatin-treated cells (Fig. 3a). Additional qRT-PCR experiments revealed that the expressions of Plk2 and Nrf2 mRNA were markedly diminished in p53-depleted NRK52E cells with cisplatin treatment, indicating that p53 was involved in the expressions of Plk2 and Nrf2 in response to cisplatin-induced AKI (Fig. 3b).

Next, we examined whether Plk2 depletion using shRNA targeting *Plk2* affects the expression of Nrf2 in cisplatin-treated NRK52E cells (Fig. 3c). Knockdown of *Plk2* did not affect the expression or stability of p53 (Fig. 3c). However, Plk2 depletion reduced the levels of both soluble and chromatin-bound Nrf2 (Fig. 3c). Nrf2 mRNA levels were downregulated in cisplatin-treated Plk2-depleted cells compared with those in cisplatin-treated shControl cells (Fig. 3d). However, p53 mRNA was not affected by the presence or absence of Plk2, which is reasonable since Plk2 is a downstream target of p53. Therefore, p53 maintains the levels of Plk2, which regulates the levels and nuclear location of Nrf2 in response to cisplatin-induced stress.

To understand the function of Plk2 in cisplatin-induced AKI, Flag-tagged Plk2 was transiently overexpressed in NRK52E cells, and then, the cells were treated with cisplatin for 48 h (Fig. 3e). The expression of Plk2 was increased in cisplatin-treated cells expressing Flag-tagged Plk2 compared with that in the control (Fig. 3e). Under the same condition, the levels of soluble and chromatin-bound Nrf2 proteins increased by treatment of cisplatin in concurrence with the changes of Flag-Plk2 protein levels (Fig. 3e). Consistent with the results of the Plk2 knockdown experiment, the levels of p53 were not altered by Flag-tagged Plk2 overexpression. The mRNA levels of Nrf2 were increased in cells expressing Flag-Plk2 and treated with cisplatin, but p53 mRNA was not altered (Fig. 3f), indicating that Nrf2 expression is regulated by Plk2, whereas p53 expression is not changed by Plk2.

### Nrf2 negatively regulates the expression of p53 and Plk2 by upregulating Mdm2

**Fig. 4:**
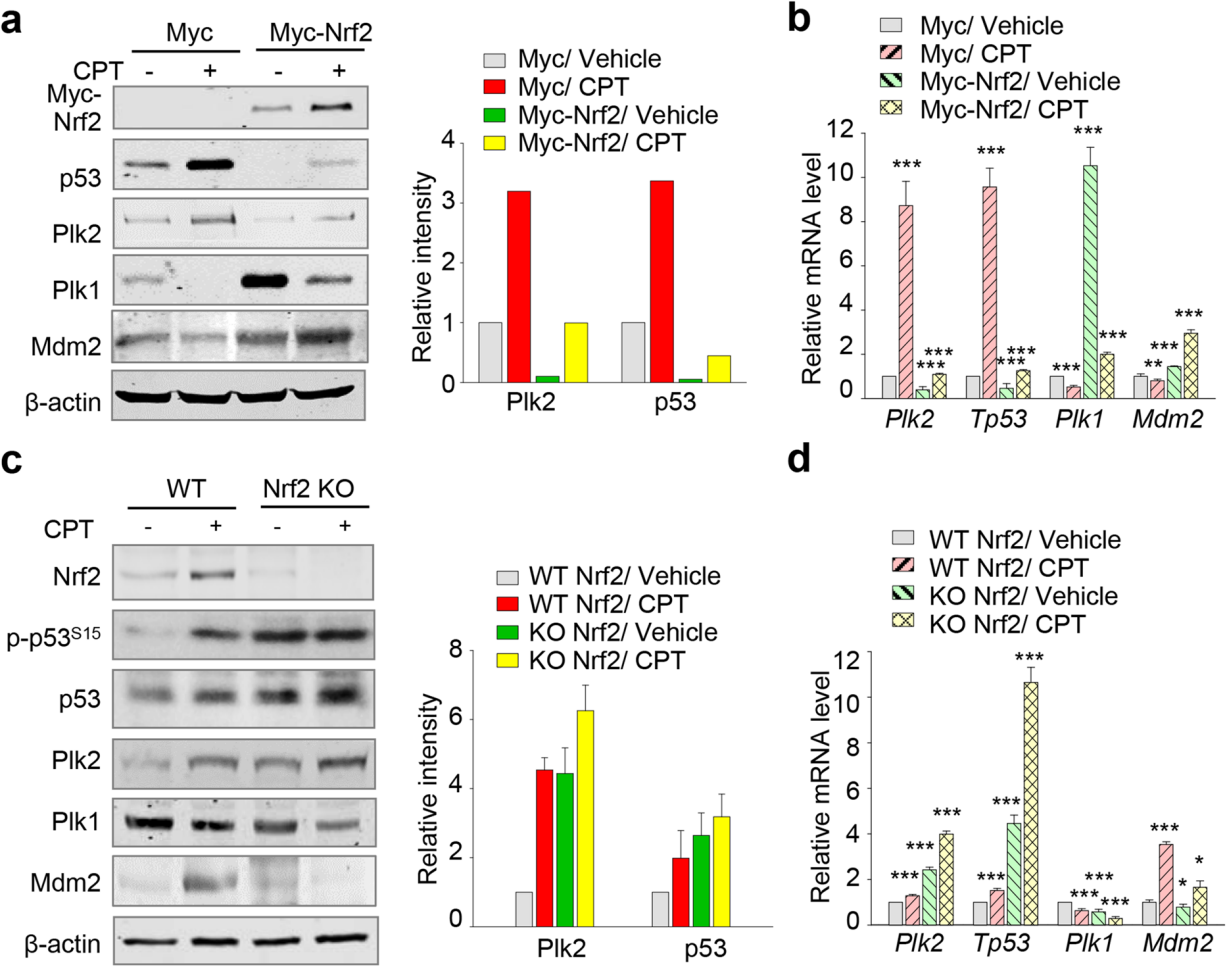
Nrf2 negatively regulates the expression of p53 and Plk2 by upregulating Mdm2. **a**–**b** Myc-tagged Nrf2 proteins were expressed in NRK52E cells. Twenty-four hours after transfection, the cells were treated with/without 30 μM cisplatin for 48 h. **a** Immunoblot analysis was performed with antibodies for Myc, p53, Plk2, Plk1, Mdm2, and β-actin. The relative band intensity values of p53 and Plk2 were analyzed using LI-COR Odyssey software. **b** qRT-PCR was performed to evaluate the mRNA levels of rat *Plk2*, *Tp53*, *Plk1*, and *Mdm2* in NRK52E cells. ***p < 0.001. **c**–**d** The kidney cortex tissues of Nrf2-/- mice were taken 3 days after a single injection of cisplatin (15 mg/kg, i.p., *n* = 4 or 5 per group). **c** Using the lysates of kidney cortex of mice, immunoblot analysis was performed. The expressions of Nrf2, p-p53 (Ser15), p53, Plk1, Plk2, Mdm2, and β-actin were measured using LI-COR Odyssey software and plotted. **d** qRT-PCR was performed to determine the mRNA levels of *Plk2*, *Tp53*, *Plk1*, and *Mdm2* in the kidney of mice treated with cisplatin. The relative expression of each gene was plotted. ***p < 0.001 compared with the vehicle of Nrf2 WT mice samples

To examine the regulation of the p53 signaling pathway by Nrf2 in cisplatin-induced AKI, Myc-tagged Nrf2 was transiently overexpressed in NRK52E cells, and then, the cells were treated with cisplatin for 48 h (Fig. 4a). Nrf2 expression downregulated the expression of p53 and its downstream factor Plk2, but it upregulated the expression of Mdm2, an E3 ligase of p53, and Plk1, a proliferating factor (Fig. 4a). mRNA levels of p53 and Plk2 were markedly downregulated in cisplatin-treated cells expressing Myc-Nrf2 compared with those in cisplatin-treated control cells (Fig. 4b). Plk1 mRNA levels were highly expressed in untreated cells expressing Myc-Nrf2 but not in cisplatin-induced cells expressing Myc-Nrf2 (Fig. 4b). Mdm2 mRNA levels were upregulated in cells expressing Myc-Nrf2 compared with those of cells expressing Myc-mock (Fig. 4b). Thus, overexpressed Nrf2 suppresses the expression of p53 and Plk2 but upregulates the expression of Mdm2 and Plk1. Overexpressed Nrf2 negatively regulated the levels of p53 and Plk2 while promoting the expression of Mdm2 and Plk1.

To understand the role of Nrf2 in the response to cisplatin-induced stress in vivo, Nrf2 KO mice were treated with 15 mg/kg cisplatin. Total and active p53 levels were highly upregulated in Nrf2 KO mice. In addition, the protein and mRNA levels of Plk2 were higher in cisplatin-treated Nrf2 KO mice than those in wild-type mice treated with cisplatin (Fig. 4c–d), indicating that the p53/Plk2 signaling pathway is more amplified in the absence of the oxidative stress responder Nrf2. Since Nrf2 is known as a transcriptional factor for Mdm2 (You et al. 2011), the levels of Mdm2 were observed in Nrf2 KO mice. As expected, the protein and mRNA levels of Mdm2 were lower in cisplatin-treated Nrf2 KO mice than those in cisplatin-treated wild-type mice (Fig. 4c–d). Together, these findings show that overexpression of Nrf2 suppresses the expression of p53 and Plk2, but depletion of Nrf2 upregulates p53 and Plk2. Consistently, the opposite pattern was observed in the levels of Mdm2. Therefore, Nrf2 negatively regulates p53 and Plk2 by upregulating Mdm2, suggesting that Nrf2 is a negative feedback regulator of p53/Plk2 signaling.

### Methotrexate-induced AKI of NRK52E cells resulted in the upregulation of Plk2 and chromatin-bound Nrf2

**Fig. 5:**
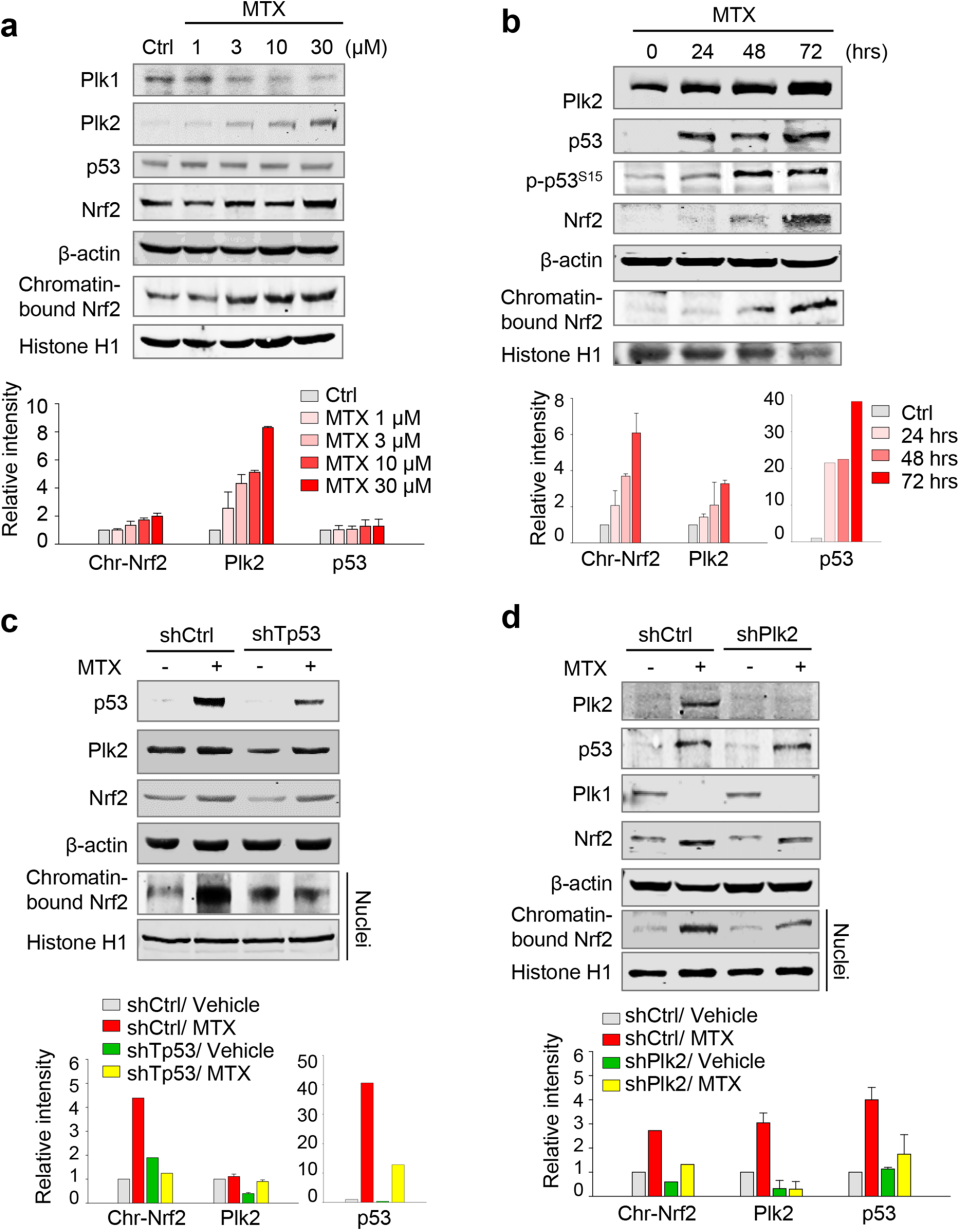
Upregulation of Plk2 and chromatin-bound Nrf2 in methotrexate-treated NRK52E cells. **a** Methotrexate (MTX) was treated at the indicated concentrations in NRK52E cells. Immunoblot was performed with whole lysate using specific antibodies for Plk1, Plk2, p53, Nrf2, Histone H1, and β-actin. The relative band intensity values were quantified and plotted. **b** NRK52E cells were treated with 30-μM methotrexate for the indicated times. Immunoblot analysis was performed for p53, p-p53 (Ser15), Plk2, Nrf2, β-actin, and Histone H1. Chromatin-bound Nrf2 and Histone H1 were detected using nuclear fractions. The relative band intensity values were quantified and plotted. **c** NRK52E cells were infected with lentivirus expressing rat *Tp53* shRNA. Twenty-four hours after infection, the cells were treated with/without 30 μM methotrexate for 48 h. Immunoblot analysis was performed with antibodies for p53, Plk2, Nrf2, β-actin, and Histone H1. The relative band intensity values of p53, Plk2, and chromatin-bound Nrf2 were quantified and plotted. **d** Twenty-four hours after infection with lentiviral Plk2 shRNA in NRK52E cells, the cells were treated with 30 μM methotrexate for 48 h. Immunoblot analysis was performed with antibodies for Plk2, p53, Plk1, Nrf2, Histone H1, and β-actin. The relative band intensity values were plotted

In cisplatin-induced AKI, p53/Plk2 signaling was activated, and thus, Nrf2 consequently translocated to chromatin in the nucleus (Figs. 1, 2, 3, and 4). To examine the activation of p53/Plk2/Nrf2 signaling in response to the AKI, methotrexate was used as another inducer for AKI (Soon and Ilchyshyn 2005; Strang and Pullar 2004). We investigated the expression of Plk2, p53, and Nrf2 mRNA and protein in NRK52E cells treated with various concentrations of methotrexate for 48 h (Fig. 5a) or at 30 μM for 24, 48, and 72 h (Fig. 5b). Protein levels of Plk2, p53, and Nrf2 were increased in a concentration-dependent and time-dependent manner in methotrexate-treated NRK52E cells (Fig. 5a–b). Consistent with the results of cisplatin-induced AKI, chromatin-bound Nrf2 protein increased after methotrexate treatment (Fig. 5a–b). Additional experiments using p53-depleted NRK52E cells showed that chromatin-bound Nrf2 was markedly decreased compared with soluble Nrf2 in methotrexate-treated and p53-depleted NRK52E cells (Fig. 5c). Since the levels of Plk2 were upregulated in AKI, Plk2 was depleted in NRK52E cells (Fig. 5d). Plk2-depleted kidney cells were treated with methotrexate, and Nrf2 levels were examined (Fig. 5d). These results showed that Plk2 depletion resulted in reduced levels of Nrf2 despite the presence of methotrexate and the levels of chromatin-bound Nrf2 were also markedly reduced (Fig. 5d), indicating that the presence of Plk2 is important for the expression and nuclear translocation of Nrf2. Thus, methotrexate-induced AKI also increased levels of Plk2, p53, and Nrf2 in a time-dependent or a concentration-dependent manner, which triggered chromatin localization of Nrf2.

### Phosphorylated Nrf2 at Ser40 by Plk2 interacts with p21^cip1^, which accelerates translocalization into the nuclei

**Fig. 6:**
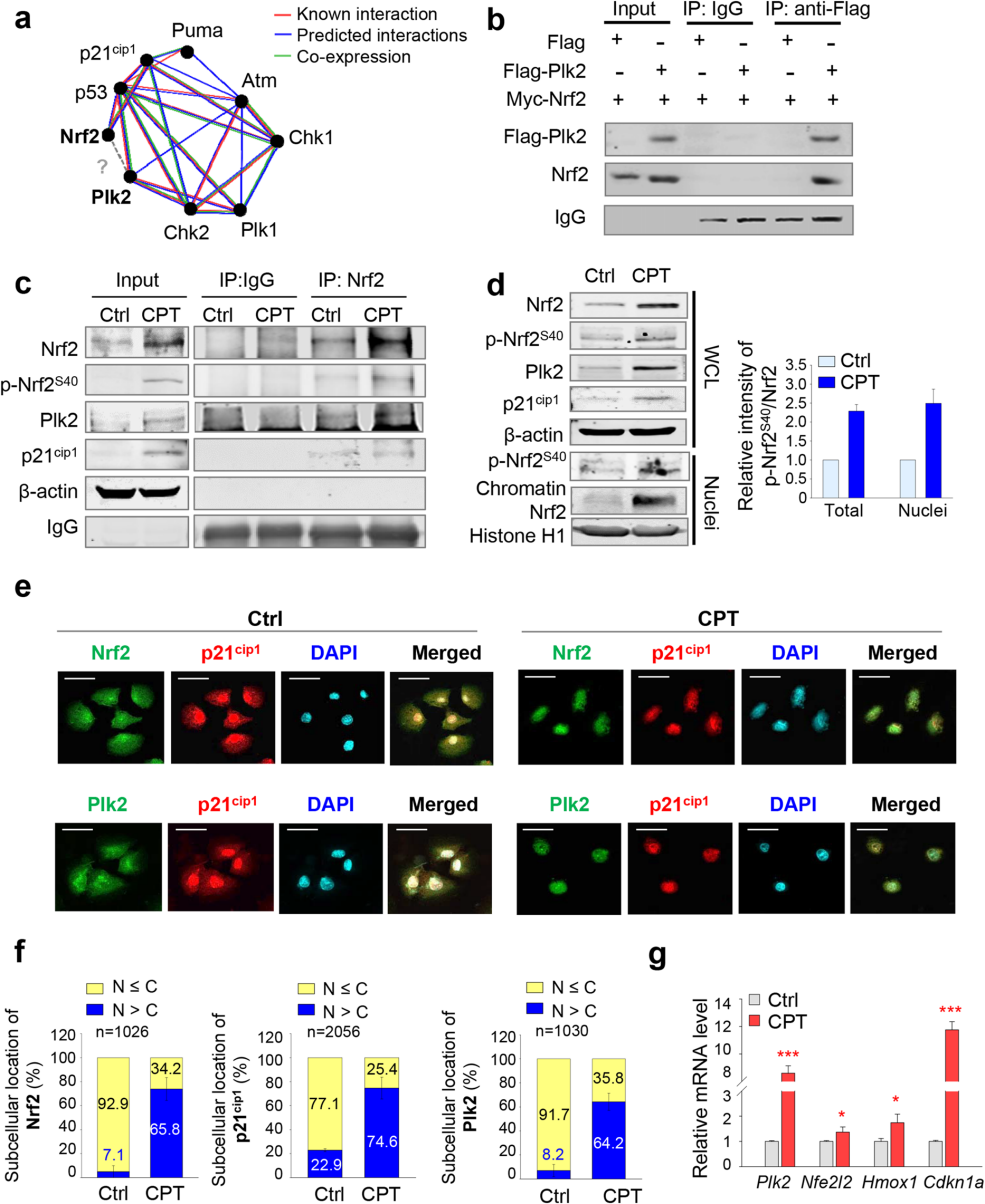
Phosphorylated Nrf2 at Ser40 by Plk2 interacts with p21^cip1^, which accelerates translocalization into the nuclei in cisplatin-induced stress of NRK52E cells. **a** The network of p53 signaling factors was extracted from the GeneMANIA database. Red line, known interaction; blue line, predicted interaction; and green line, co-expression. **b** Flag-tagged Plk2 and/or Myc-tagged Nrf2 were expressed in HEK293 cells. Immunoprecipitation was performed with anti-Flag antibody. Immunoblot was performed with anti-Plk2 and anti-Nrf2 antibodies. **c** NRK52E cells were grown for 48 h in the presence of 30-μM cisplatin. Immunoprecipitation was performed with anti-Nrf2 antibody, and immunoblot was performed using specific antibodies for Plk2, Nrf2, p-Nrf2 (S40), p21^cip1^, and β-actin. **d** Cisplatin at 30 μM was treated to NRK52E cells for 48 h. Immunoblot was performed with whole lysates or nuclear fractions using specific antibodies for Plk2, Nrf2, p-Nrf2 (S40), p21^cip1^, β-actin, and Histone H1. **e** Cells were grown for 48 h in the presence or absence of 30-μM cisplatin. The cells were fixed with 4% paraformaldehyde and stained for Nrf2 or Plk2 (green), p21^cip1^ (red), and DAPI (blue). Bars, 25 μM. **f** The population of cells showing the indicated subcellular location of Nrf2, Plk2, and p21.^cip1^ of Fig. 6e was quantified in the presence or absence of 30-μM cisplatin. **g** qRT-PCR was performed using NRK52E cells in the presence or absence of 30-μM cisplatin. Relative mRNA expression levels of *Plk2*, *Nfe2l2*, *Hmox1*, and *Cdkn1a* were determined and plotted. *p < 0.05; ***p < 0.001 compared with control cells

We previously found that Plk2 phosphorylates Nrf2, translocating Nrf2 into the nuclei and upregulating its transcriptional activity in hepatocellular carcinoma (Joo et al. 2019). To determine whether these events occur in kidney cells in response to acute injury, we examined the phosphorylation of Nrf2 and its translocation into the nuclei. First, the interaction between Nrf2 and p53-related factors including Plk2 was assessed using the GeneMANIA database (Warde-Farley et al. 2010) (Fig. 6a). The network showed the interaction between Nrf2 and p53 as well as between Plk2 and p53, but the interaction between Nrf2 and Plk2 was unclear in the database (even though our results demonstrated this interaction) (Fig. 6a). The relations between Nrf2 and Plk2 were investigated to understand how Plk2 contributes to the function of Nrf2 in cisplatin-induced AKI. An interaction between exogenously expressed Plk2 and Nrf2 was observed in human embryonic kidney cells after transient expression of Flag-tagged Plk2 along with Myc-tagged Nrf2 (Fig. 6b). Previously, we found that Plk2 phosphorylates Nrf2 at Ser40 and Ser215 in vitro, as determined by LC–MS/MS analysis and in vitro kinase assay using peptidomimetic methods or site-directed mutagenic Nrf2 (Joo et al. 2019) (Supplementary Fig. 1). An in vitro Plk2 kinase assay using human Nrf2 having alanine substitutes at both Ser40 and Ser215 residues showed that the level of phosphorylation was markedly lower in a double mutant (S40A/S215A; AA) of Nrf2 than that in wild-type Nrf2 or single alanine substitute of Nrf2, indicating that Nrf2 is phosphorylated by Plk2 at Ser215 and Ser40 (Supplementary Fig. 1). In addition, their interaction and the phosphorylation of Nrf2 at Ser40 were observed in NRK52E cells treated with cisplatin by immunoprecipitation assay (Fig. 6c). The levels of interaction between endogenous Nrf2 and Plk2 were higher in cisplatin-treated cells than those in control cells. Of note, Plk2 interacted with Nrf2 phosphorylated at Ser40 in cisplatin-treated NRK52E cells. Moreover, we observed the interaction between Nrf2 and p21^cip1^, consistent with a study that reported p21^cip1^ as an interacting factor of Nrf2 (Chen et al. 2009). As shown in Fig. 6c, Nrf2 interacted with p21^cip1^ in cisplatin-treated kidney cells.

The subcellular locations of p-Nrf2^S40^ were observed using fractionation assay and immunostaining analysis (Fig. 6d–f). In the fractionation assay, the nuclear levels of Nrf2 and p-Nrf2^S40^ were highly upregulated in cisplatin-treated cells compared with those of control cells (Fig. 6d). The relative intensity of p-Nrf2^S40^/Nrf2 was higher in the nuclear fraction compared with that in the total fraction. Next, immunofluorescence staining was performed to examine the translocation of Nrf2 into the nucleus in cells treated with cisplatin (Fig. 6e). In control cells, Nrf2 and p21^cip1^ proteins were located in both cytoplasm and nucleus. In cisplatin-treated NRK52E cells, Nrf2 and p21^cip1^ proteins translocated into the nucleus and showed colocalization in the nuclei (Fig. 6e). Additionally, the colocalization of Plk2 and p21^cip1^ was observed. The percentages of nuclear Nrf2-, p21^cip1^-, and Plk2-positive cells were approximately 65.8%, 74.6%, and 64.2%, respectively, in cisplatin-treated NRK52E cells (Fig. 6f), indicating that cisplatin-induced AKI triggers the nuclear translocation of Nrf2, p21^cip1^, and Plk2.

To understand the function of nuclear Nrf2 in cisplatin-induced AKI, the levels of target genes of Nrf2 including *Hmox1* were examined after cisplatin treatment for 48 h (Fig. 6g). qRT-PCR analysis revealed that the levels of target gene *Hmox1* were upregulated approximately 1.7 times compared with that of controls. In addition, the mRNA expression levels of *Nfe2l2*, *Plk2*, and *Cdkn1a* (which encodes p21^cip1^) increased 1.4 times, 8.6 times, and 11.8 times, respectively, by treatment of cisplatin in NRK52E cells (Fig. 6g). These results indicated that Nrf2 was translocated into the nucleus when AKI was triggered by stress-inducing agents such as cisplatin. Taken together, these findings indicate that Plk2-mediated phosphorylation of Nrf2 regulates its localization to the nuclei in response to the cisplatin-induced stress.

### Plk2-dependent phosphorylation of Nrf2 facilitates anti-oxidative and anti-inflammatory response in cisplatin-induced stress of rat kidney NRK52E cells

**Fig. 7:**
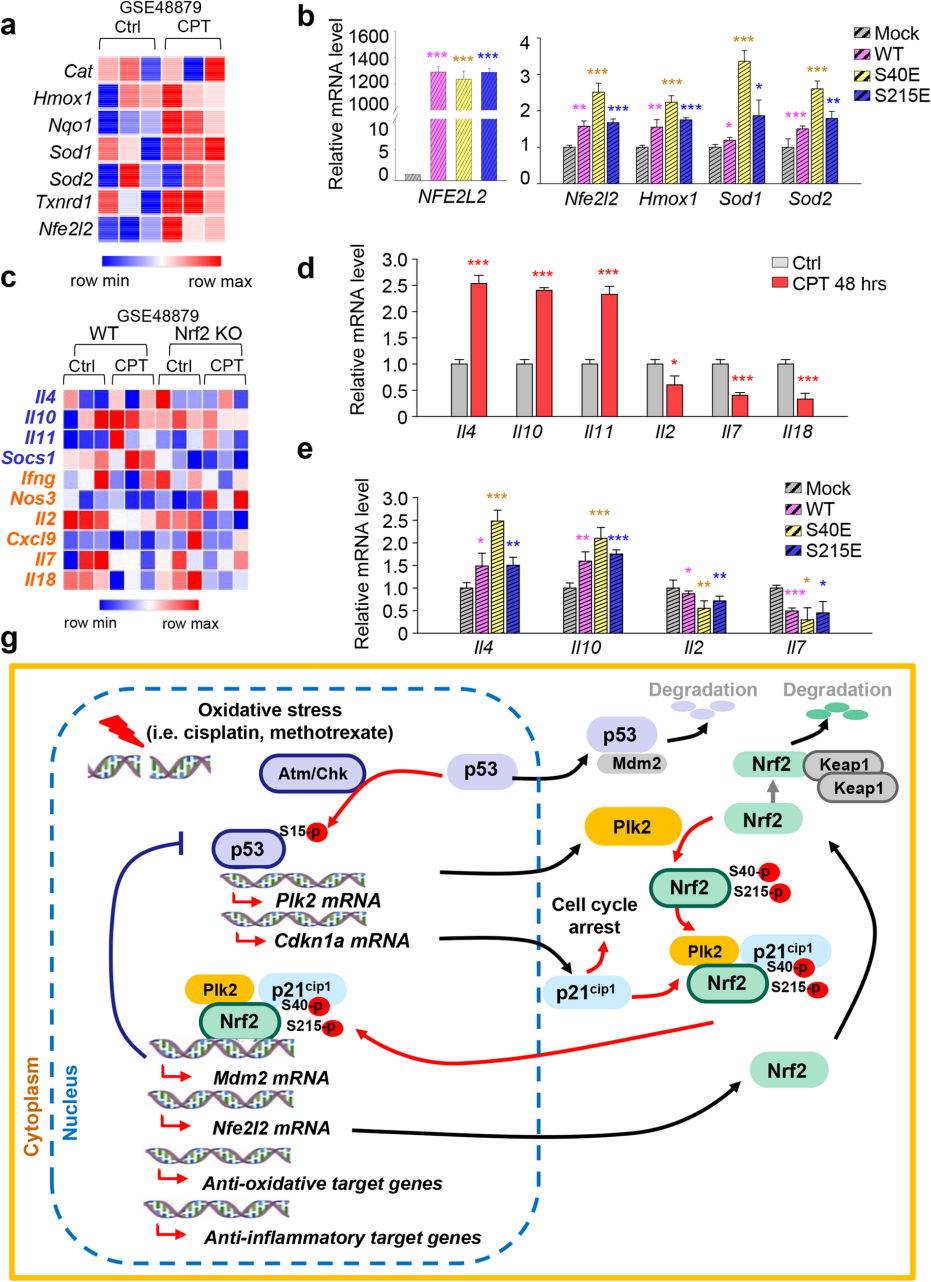
Plk2-dependent phosphorylation of Nrf2 facilitates the expression of anti-oxidative and anti-inflammatory genes in rat kidney NRK52E cells. **a** A heatmap analysis was performed for *Cat*, *Hmox1*, *Nqo1*, *Sod1*, *Sod2*, *Txnrd1*, and *Nfe2l2* using a published transcriptome dataset (GSE48879) from cisplatin-treated mice. **b** qRT-PCR was performed for human *NFE2L2*, rat *Nfe2l2*, *Hmox1*, *Sod1*, and *Sod2* in NRK52E cells expressing Myc-tagged mock, wild-type (WT) human Nrf2, S40E, or S215E mutant. *NFE2L2* is for the exogenous Myc-tagged human Nrf2*.* *p < 0.05; **p < 0.01; ***p < 0.001** c** A heatmap analysis was performed for *Il4*, *Il10*, *Il11*, *Il2*, *Il7*, *Il18*, *Socs1*, *Ifng*, *Nos3*, and *Cxcl9* using a published transcriptome dataset (GSE48879) from cisplatin (CPT)–treated wild-type (WT) and Nrf2 KO mice. **d** qRT-PCR was performed for *Il4*, *Il10*, *Il11*, *Il2*, *Il7*, and *Il18* in NRK52E cells treated with 30-μM cisplatin for 48 h. *p < 0.05; ***p < 0.001. **e** qRT-PCR was performed for *Il4*, *Il10*, *Il2*, and *Il7* in NRK52E cells expressing mock, wild type (WT), S40E, and S215E of Nrf2*.* *p < 0.05; **p < 0.01; ***p < 0.001 compared with control cells expressing myc-mock. **f** A plausible model of Nrf2 activation signaling: p53-mediated upregulation of Plk2 and p21^cip1^ induces the activation of Nrf2 through phosphorylation and recruitment to the nuclei for the expression of target genes related to anti-oxidative and anti-inflammatory signaling

Next, we investigated the function of phosphorylation of Nrf2 in cisplatin-induced AKI. First, we examined the expression of target genes of Nrf2 in cisplatin-treated mice in vivo using a published transcriptome of cisplatin-treated mice (GSE48879). In cisplatin-treated mice, the expressions of *Hmox1*, *Nqo1*, *Sod1*, *Sod2*, and *Txnrd1*, well-known Nrf2 target genes, were all upregulated, as compared with controls (Fig. 7a). The expression of *Cat* was not affected. It has been reported that Nrf2 can regulate its expression by directly binding to the ARE-L1/2 regions of the Nrf2 promoter as well (Hayes and Dinkova-Kostova 2014; Kwak et al. 2002). To investigate the effects of Nrf2 phosphorylation on the expression of target genes or Nrf2 itself in cisplatin-induced AKI, phosphomimetics of Nrf2 at Ser40 and Ser215 were expressed in NRK52E cells (Fig. 7b). qRT-PCR assay was performed to evaluate mRNA levels. When the expressions of exogenous human *NFE2L2* were similar in NRK52E cells expressing wild type or phosphomimetics of Nrf2, the levels of *rat Nfe2l2* in cells expressing wild-type Nrf2 were upregulated up to approximately 1.6 times, compared with those of mock. In addition, expression of S40E Nrf2 increased the levels of *rat Nfe2l2* up to 2.5 times, compared with those of mock. The effects of S215E and wild-type Nrf2 on *rat Nfe2l2* expression were similar (Fig. 7b). Furthermore, in cells expressing phosphomimetics at Ser40, the levels of rat *Hmox1*, *Sod1*, and *Sod2* were upregulated 2.2 times, 3.4 times, and 2.6 times, respectively, compared with those of control NRK52E cells. The gene transcript levels slightly increased in cells expressing S215E compared with those expressing the wild type (Fig. 7b). These data indicate that the phosphorylation of Nrf2 is necessary for the expression of Nrf2 itself and target genes, especially at Ser40 compared with Ser215.

To determine which genes are expressed in response to cellular stress related to Nrf2, we examined several cytokines, since Nrf2 and its downstream factors including *Hmox1* regulate inflammatory signals (Ma 2013). First, the expressions of the pro-inflammatory and anti-inflammatory cytokines were reanalyzed using microarray data (GSE48879) from cisplatin-treated wild-type mice and Nrf2 KO mice. The levels of anti-inflammatory cytokines (*Il4*, *Il10*, *Il11*, and *Socs1*) and pro-inflammatory cytokines (*Ifng*, *Nos3*, *Cxcl9*, *Il2*, *Il7*, and *Il18*) were displayed with a heatmap (Fig. 7c). In wild-type mice, cisplatin treatment increased the average mRNA levels of anti-inflammatory cytokines but reduced the average levels of pro-inflammatory cytokines compared with levels in untreated wild-type control mice (Fig. 7c), indicating that cisplatin treatment induces anti-inflammatory signals. In Nrf2 KO mice, cisplatin-induced AKI downregulated the expression of anti-inflammatory cytokines such as *Il4*, *Il10*, and *Socs1*. However, pro-inflammatory cytokines including *Ifng*, *Cxcl9*, *Il2*, *Il7*, and *Il18* were not upregulated in cisplatin-treated Nrf2 KO mice compared with levels in untreated Nrf2 KO mice, indicating that Nrf2-mediated regulation is more directly related to anti-inflammatory signals. When the protein levels of anti-inflammatory cytokines IL4 and IL10 were observed, they were lower in kidney tissues of Nrf2 KO mice treated with cisplatin than those in wild-type mice treated with cisplatin (Supplementary Fig. 2). Based on the microarray data and immunoblot analysis, we next examined the mRNA levels of anti-inflammatory cytokines (*Il4*, *Il10*, and *Il11*) and pro-inflammatory cytokines (*Il2*, *Il7*, and *Il18*) in cisplatin-treated NRK52E cells (Fig. 7d). Cisplatin treatment upregulated the mRNA levels of *Il10*, *Il4*, and *Il11* by approximately 2.4 times, 2.5 times, and 2.3 times, respectively. However, it downregulated the levels of *Il2*, *Il7*, and, *Il18* by approximately 0.5 times, 0.4 times, and 0.3 times, respectively (Fig. 7d), indicating that cisplatin-induced AKI results in the activation of anti-inflammatory signals.

To investigate the effects of phosphorylated Nrf2 on the expression of cytokines, mRNA levels of *Il4*, *Il10*, *Il2*, and *Il7* were examined in rat kidney NRK52E cells expressing phosphomimetics of Nrf2. The mRNA levels of *Il4* and *Il10* increased approximately 2.1-fold and 2.5-fold, respectively, in the cells expressing phosphomimetics of Nrf2 at Ser40 compared with those of controls (Fig. 7e). The levels of pro-inflammatory cytokines *Il2* and *Il7* were downregulated by approximately 0.7- and 0.5-fold in the cells expressing S40E compared with those of mock (Fig. 7e). Changes of these genes in NRK52E cells expressing wild-type and S215E Nrf2 were similar to each other, which was less than those expressing S40E Nrf2 (Fig. 7e). These findings indicate that the phosphorylation of Nrf2 at Ser40 by Plk2 induces the expression of anti-inflammatory cytokines including *Il4*, *Il10*, and *Il11* as well as antioxidant stress genes such as *Hmox1*, *Sod1*, and *Sod2*.

## Discussion

We have reported the anti-oxidative and anti-inflammatory p53/Plk2/Nrf2 signaling axis in response to AKI. Our findings shown here in this study include the following: (1) AKI increases the expressions of Plk2, p53, and Nrf2, which consequently upregulates the levels of nuclear chromatin-bound Nrf2 in mouse and rat kidney NRK52E cells; (2) regulation of the levels of Plk2 by p53, in turn, activates Nrf2 and its nuclear location in response to cisplatin- or methotrexate-induced stress in NRK52E cells; (3) Nrf2 negatively regulates the levels of p53 and Plk2 by upregulating Mdm2, functioning as a negative feedback loop; (4) Plk2-mediated phosphorylation of Nrf2 at Ser40 accelerates the interaction with p21^cip1^ and translocation into the nuclei during AKI; and (5) Plk2-dependent phosphorylation of Nrf2 facilitates anti-oxidative and anti-inflammatory responses in cisplatin-induced stress of rat kidney NRK52E cells (Fig. 7f). Taken together, these data suggest that Plk2 is an anti-oxidative and anti-inflammatory regulator by phosphorylating Nrf2, which then triggers translocalization of Nrf2 into the nuclei and expression of anti-oxidative and anti-inflammatory gene products for the protection of the cells from AKI (Fig. 7g).

Plk2 is recognized as a tumor suppressor in several cancers (Burns et al. 2003; Coley et al. 2012; Matthew et al. 2007; Pellegrino et al. 2010; Smith et al. 2006; Zhao et al. 2015). Plk2 is epigenetically inactivated in malignant lymphomas (Smith et al. 2006), and silencing of Plk2 enhances tumor growth in non–small cell lung carcinoma, ovarian carcinoma, and gastric cancer (Coley et al. 2012; Matthew et al. 2007; Zhao et al. 2015). Plk2 is frequently silenced in a methylation-dependent manner in several B cell lymphomas and primary lymphomas (Benetatos et al. 2011; Smith et al. 2006). In contrast, Plk2 is also reported as an oncogenic factor based on its overexpression and tumor-promoting effects in colorectal cancer, bladder cancer, and pancreatic cancer (Kothari et al. 2013; Ou et al. 2016; Tan et al. 2010). Even though the function of Plk2 in cancer may depend on tissue-specific characteristics, most studies on Plk2 have focused on defense mechanisms in response to oxidative stress in cellular damage. Plk2 works as an activator of the anti-oxidative pathway in response to mitochondrial defects in the presence of oxidative stress (Li et al. 2014). It functions as a potent antioxidant molecule through GSK3β phosphorylation, which affects the translocalization of Nrf2 (Li et al. 2014). In the present study, we found that Plk2 directly phosphorylates Nrf2 to elicit translocalization in response to cellular stress in kidney cells. Phosphorylation of Nrf2 at Ser40 by Plk2 accelerated the interaction with p21^cip1^ and translocation into the nuclei in AKI, as evidenced by immunoprecipitation and immunofluorescence. In addition, the expression of phosphomimetics of Nrf2 at Ser40 facilitated the expression of anti-oxidative and anti-inflammatory factors as well as *Nfe2l2* itself in kidney NRK52E cells. These findings indicate that Plk2 plays a role in the defense mechanism from oxidative stress and DNA damage in AKI. Of note, Plk2 induced the expression of Nrf2; overexpression of Plk2 upregulated the levels of Nrf2 protein and mRNA, whereas depletion of Plk2 using shRNA downregulated the expression of Nrf2 as shown in Fig. 3. Since Nrf2 can regulate its expression by directly binding to the ARE-L-1/2 region of the Nrf2 promoter (Hayes and Dinkova-Kostova 2014; Kwak et al. 2002), the stabilized Nrf2 by Plk2-mediated phosphorylation and formation of Plk2/Nrf2/p21^cip1^ complex translocates into the nuclei. Consequently, the Nrf2 would bind to the ARE-L1/2 located at the promoter of *Nrf2* for its own gene induction. Moreover, our result demonstrates that expression of S40E Nrf2 increased the levels of *rat Nfe2l2*, compared with those of mock, wild type, and S215E of Nrf2, indicating that the phosphorylation of Nrf2 by Plk2 is important for the expression of Nrf2 itself, especially at Ser40 compared with Ser215.

However, overexpression of Nrf2 suppressed the expression of p53 and its downstream target Plk2 (see Fig. 4). Nrf2 KO resulted in the upregulation of p53 and Plk2 with or without oxidative stress, indicating that high levels of Nrf2 negatively regulate the expression of p53 and Plk2 for homeostasis in kidney cells. Since Nrf2 is known as a transcriptional factor for Mdm2 (You et al. 2011), an E3 ubiquitin-protein ligase for degradation of p53, we observed the levels of Mdm2 in Nrf2-overexpressed or Nrf2-depleted cells, to investigate how Nrf2 negatively controls the levels of p53 and Plk2. As shown in Fig. 4, the levels of Mdm2, an E3 ligase of p53, were higher in cells expressing Myc-Nrf2 than those of control, which would downregulate the levels of p53 through the degradation pathway. Our result also showed that Mdm2 levels were lower with the higher levels of p53 in the kidney cortex of Nrf2 KO mice, compared to those of WT control. Therefore, it is highly likely that Nrf2 negatively regulates p53 and its target Plk2 by upregulating Mdm2 in kidney cells.

Nrf2 functions as an anti-inflammatory regulator as well as anti-oxidative factor through the expression of antioxidant genes that encode the factors that defend cells from injurious stimuli through their anti-inflammatory effects (Boyanapalli et al. 2014; Ma 2013). Nrf2 triggers the expression of downstream anti-oxidative genes, such as heme oxygenase1 (*Hmox1*), NAD(P)H quinone oxidoreductase (*Nqo1*), superoxide dismutase (*Sod1*, *Sod2*), and phase II enzymes, upon cellular stress as a cellular stress defense mechanism (Kobayashi and Yamamoto 2006; Ma 2013). Nrf2 is critical for protecting cells from the renal toxicity induced by cisplatin, an anticancer drug, through transcriptional activation of microRNA-125b (Joo et al. 2013). Additionally, our recent study revealed that Nrf2-activating lncRNA functions as a cell fate regulator by p53-dependent Nrf2 activation and that Plk2 forms a complex with Nrf2 under survival conditions from oxidative stress in hepatocellular cancer (Joo et al. 2019). Although the importance of Nrf2 and Plk2 in cellular defense in response to oxidative stress has been addressed, the basis as to how Plk2 and Nrf2 protect the kidney cells from AKI had been unknown. In this study, we report that in response to AKI, the activation of Atm and p53 elicits the expression of Plk2/p21^cip1^, which activates Nrf2 through phosphorylation by Plk2 and recruitment of Nrf2 by p21^cip1^ into the nuclei for expression of anti-oxidative and anti-inflammatory factors in response to AKI. The interaction between p21^cip1^ and Nrf2 increases the stability of Nrf2 by causing dissociation of Nrf2 with Keap1 (Chen et al. 2009). p53-dependent upregulation of Plk2 phosphorylates Nrf2 at Ser40, facilitating its translocalization into the nuclei and the expression of downstream factors related to anti-oxidative and anti-inflammatory genes in response to oxidative stress of AKI. Even though the phosphorylation of Nrf2 at Ser40 by PKCδ was previously identified, PKCδ may be subjected to degradation in response to DNA damage (Basu et al. 2001; Huang et al. 2000). In response to oxidative stress of AKI, Plk2 operates as an Nrf2 kinase in a p53-dependent signaling pathway. Moreover, Plk2-mediated phosphorylation of Nrf2 regulates inflammation in AKI. Cisplatin-induced AKI or expression of phosphomimetics of Nrf2 at Ser40 triggers the upregulation of anti-inflammatory cytokines and downregulation of pro-inflammatory cytokines. Accumulating evidence supports that Nrf2-mediated signaling has a protective function against oxidative stress and inflammation (Boyanapalli et al. 2014; Kobayashi et al. 2016; Kobayashi and Yamamoto 2006; Ma 2013). Nrf2 silencing using RNA interference upregulated the transcriptional levels of pro-inflammatory cytokines in LPS-induced inflammatory conditions (Kobayashi et al. 2016). Since the transcriptional regulation of Nrf2 is based on its nuclear location, these reports support that Plk2-mediated phosphorylation of Nrf2 may be the critical process for the anti-inflammatory function as well as anti-oxidative function in response to AKI.

Interestingly, our model may be applicable to the ischemic AKI model. By analysis of the published data from the ischemic AKI model developed with unilateral renal ischemia–reperfusion injury (GSE192883), the relative gene expression of *Nfe2l2*, *Plk2*, and *Cdkn1a* was upregulated (Supplementary Fig. 3). Additionally, target genes of Nrf2 including *Hmox1*, *Txnrd1*, and *Il11* for anti-oxidation and anti-inflammation were upregulated at similar time points (at 28 and 30 min, unilateral clamping) (Supplementary Fig. 3). Based on the results, this regulatory mechanism on the Nrf2 pathway is also applicable to the ischemic AKI model.

Nrf2 mutations were detected in some cancers by genetic analysis (Taguchi et al. 2011; Wang et al. 2008; Yamadori et al. 2012). It has been reported that the mutations are related to enhancing its activity and resistance to the standard chemotherapy of cancer. When AKI occurs in cisplatin-treated cancer patients, which Nrf2 modulators between activators and inhibitors would be better to improve the AKI? Cisplatin treatment increased the levels of Nrf2 in cisplatin-treated patients having wild-type Nrf2, which protects them from the oxidative stress induced by cisplatin. If patients have wild-type Nrf2, the Nrf2 activator might be useful to treat AKI during the administration of cisplatin. However, if the patients have mutant Nrf2 with high activity, the degree of AKI would be lower than that of patients having wild-type Nrf2. Therefore, to determine what is useful between Nrf2 activator or inhibitor to the patients having AKI during the administration of cisplatin, the genetic analysis of patients would be needed. Because the causal genes and factors for individual cancer are diverse, it would be needed to check which genetic problem is the main factor for the patients. Although the function of Nrf2 in cancer remains unresolved, based on the analysis of the genetic background of patients, Nrf2 modulators may be of help to treat AKI or/and cancer.

## Supplementary Information

Below is the link to the electronic supplementary material.

Supplementary file1 (DOCX 198 KB)
