# Supplementary material for: Plk2-mediated phosphorylation and translocalization of Nrf2 activates anti-inflammation through p53/Plk2/p21cip1 signaling in acute kidney injury
Source: Cell Biol Toxicol. 2022 Jul 16;39(4):1509–29. doi: 10.1007/s10565-022-09741-1 (PMC10425522; doi:10.1007/s10565-022-09741-1)

**Supplemental Information**

**Plk2-mediated phosphorylation and translocalization of Nrf2 activates anti-inflammation through p53/Plk2/p21^cip1^ signaling in acute kidney injury**

Da-Eun Kim^1^, Hye Eun Byeon^1^, Dae-Hoon Kim^1^, Sang Geon Kim^2,3*^, and Hyungshin Yim^1*^

^1^Department of Pharmacy, College of Pharmacy, **Institute of Pharmaceutical Science and Technology,** Hanyang University, Ansan, Gyeonggi-do 15588, Korea

^2^College of Pharmacy and Integrated Research Institute for Drug Development, Dongguk University-Seoul, Goyang-si, Gyeonggi-Do 10326, Korea

^3^College of Pharmacy, Seoul National University, Gwanakro 599, Seoul 08826, Korea

**Supplemental Table 1. Sequences of forward (F) and reverse (R) primers used for RT-PCR amplification.**

| Target Gene | Primer | Sequences |
| --- | --- | --- |
| Rat *Hmox1* | Forward  Reverse | 5’- CCACCAAGTTCAAACAGCTC -3’  5’- TTTGTGTTCCTCTGTCAGCA -3’ |
| Rat *Nfe2l2* | Forward  Reverse | 5’- CGGAAAACAAGCAGCAGGCT -3’  5’- CGACTGACTAATGGCAGCAGAG -3’ |
| Rat *Plk2* | Forward  Reverse | 5’- CACCACCATCATCACCATTC-3’  5’-TCGTAACACTTTGCAAATCCA-3’ |
| Rat *Cdkn1a* | Forward  Reverse | 5’- TGTTCCACACAGGAGCAAAG -3’  5’- AACACGCTCCCAGACGTAGT -3’ |
| Rat *Sod1* | Forward  Reverse | 5’- AAAGGACGGTGTGGCCAATG -3’  5’- TCCACCTTTGCCCAAGTCAT -3’ |
| Rat *Sod2* | Forward  Reverse | 5’- TTACGACTCAGGTTGCTCTT -3’  5’- CAGACACGGCTGTTAATTTC -3’ |
| Rat *Il10* | Forward  Reverse | 5’- CCACATGCTCCGAGAGCTGA -3’  5’- TCTTCACCTGCTCCACTGCC -3’ |
| Rat *Il4* | Forward  Reverse | 5’- CGAGATGTTTGTACCAGACG -3’  5’- GCTTTCCAGGAAGTCTTTCA -3’ |
| Rat *Il11* | Forward  Reverse | 5’- CACAATCTGGACTCCCTACC -3’  5’- AGAGCTGTAAGCGACGAAGT-3’ |
| Rat *Il2* | Forward  Reverse | 5’- AGTGCCTGGAAAATGAACTC -3’  5’- CTCCTCAGAAATTCCACCAC -3’ |
| Rat *Il7* | Forward  Reverse | 5’- TCAGCATCAATCAACTGGAC -3’  5’- TGTGCCGTCTGAAACTCTTA -3’ |
| Rat *Il18* | Forward  Reverse | 5’- CACTTTGGCAGACTTCACTG -3’  5’- ATCCTTCCATCCTTCACAGA -3’ |
| Rat *Mdm2* | Forward  Reverse | 5’ - GATGGCGTAAGTGACCATTC -3’  5’ - TGTGACCCGATAGACCTCAT -3’ |
| Mouse *Nfe2l2* | Forward  Reverse | 5’- CCTTCAGCAGCATCCTCTC -3’  5’- GCTTAAAGTAGCAGGTGAGGG -3’ |
| Mouse *Plk2* | Forward  Reverse | 5’- AGTGTCGATAACCCAGCAGC -3’  5’- CGAAGGACTCTTGCCACTGT -3’ |
| Mouse *Tp53* | Forward  Reverse | 5’- CCGTGTTGGTTCATCCCTGTA -3’  5’- TTTTGGATTTTTAAGACAGAGTCTTTGTA -3’ |
| Mouse *Plk1* | Forward  Reverse | 5’- CGAGGATCTGGAGGTGAAAA -3’  5’- TCTCTTTTAGGCACGAGGTC -3’ |
| Mouse *Mdm2* | Forward  Reverse | 5’ - GATGGCGTAAGTGACCATTC -3’  5’ - TGTGACCCGATAGACCTCAT -3’ |
| Human *Nfe2l2* | Forward  Reverse | 5’ - CCTTCAGCAGCATCCTCTC -3’  5’ - GCTTAAAGTAGCAGGTGAGGG -3’ |

**Supplementary Fig. 1. Phosphorylation of Nrf2 by Plk2 at Ser40 and Ser215 residues *in vitro.*** Plk2 kinase assay was performed with a GST-tagged Plk2 purified from baculovirus-infected cells, radioactive ATP, and GST-tagged wild-type (WT), S40A, S215A, S40A/S215A (AA) mutants of Nrf2.


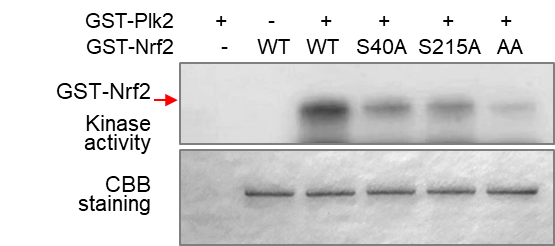


**Supplementary Fig. 2. Downregulation of IL10 and IL4 in cisplatin-treated in K/O of Nrf2 mice*.*** The kidney cortex tissues of Nrf2-/- mice were taken 3 days after a single injection of cisplatin (15 mg/kg, i.p., n=4 or 5 per group). Using the lysates of kidney cortex of mice, immunoblot analysis was performed. The expressions of Nrf2, IL4, IL10, and β-actin were measured.


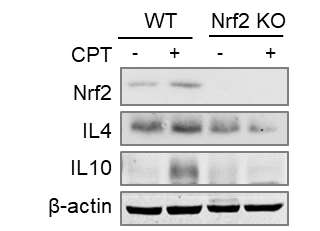


**Supplementary Fig. 3. Upregulation of *Nfe2I2*, *Plk2*, *Cdkn1a,* and target genes of Nrf2 in the ischemic AKI model.** A heatmap analysis of *Nfe2l2*, *Plk2*, *Cdkn1a,* and target genes of Nrf2 including *Hmox1*, *Nqo1*, and *Txnrd1* of the published data from the ischemic AKI model developed with unilateral renal ischemia-reperfusion injury (GSE 192883). The Ischemic AKI model was developed with unilateral renal ischemia-reperfusion injury. The left kidney was clamped for 16, 18, 20, 22, 24, 26, 28, and 30 minutes.


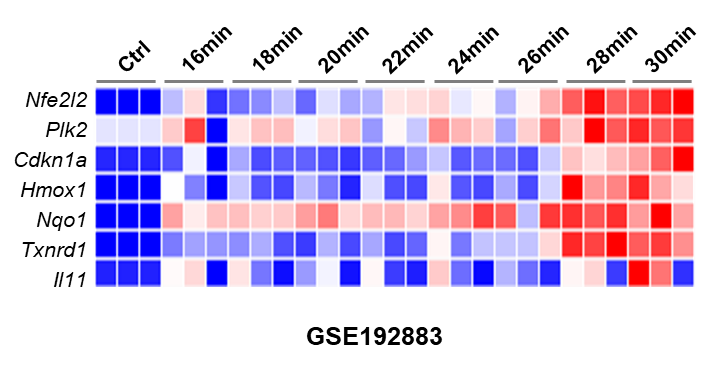

Supplement: Supplementary file 1 — Supplementary file1 (DOCX 198 KB) [file 10565_2022_9741_MOESM1_ESM.docx]
